# An Ash1-Like Protein MoKMT2H Null Mutant Is Delayed for Conidium Germination and Pathogenesis in* Magnaporthe oryzae*

**DOI:** 10.1155/2016/1575430

**Published:** 2016-09-26

**Authors:** Zhaojun Cao, Yue Yin, Xuan Sun, Jun Han, Qing peng Sun, Min Lu, Jinbao Pan, Weixiang Wang

**Affiliations:** Beijing Key Laboratory of New Technique in Agricultural Application College of Plant Science and Technology, Beijing University of Agriculture, Beijing 100026, China

## Abstract

Ash1 is a known H3K36-specific histone demethylase that is required for normal Hox gene expression and fertility in* Drosophila* and mammals. However, little is known about the expression and function of the fungal ortholog of Ash1 in phytopathogenic fungus* Magnaporthe oryzae*. Here we report that MoKMT2H, an Ash1-like protein, is required for conidium germination and virulence in rice. We obtained* MoKMT2H* null mutant (Δ*MoKMT2H*) using a target gene replacement strategy. In the Δ*MoKMT2H *null mutants, global histone methyltransferase modifications (H3K4me3, H3K9me3, H3K27me3, and H3K36me2/3) of the genome were unaffected. The Δ*MoKMT2H* mutants showed no defect in vegetative hyphal growth, conidium morphology, conidiation, or disease lesion formation on rice leaves. However, the* MoKMT2H* deletion mutants were delayed for conidium germination and consequently had decreased virulence. Taken together, our results indicated that MoKMT2H plays an important role in conidium germination during appressorium formation in the rice blast fungus and perhaps other pathogenic plant fungi.

## 1. Introduction

Covalent histone modifications, such as methylation and acetylation, provide key epigenetic information in transcriptional regulation and chromatin structure organization for functional responses [1, 2]. The vast array of such modifications gives enormous potential for functional responses. The timing of the appearance of a modification will depend on the signaling conditions within the cell. Histone methylation is catalyzed by histone methyltransferases (HMTs) and provides an excellent epigenetic mechanism for the stable transfer of gene expression profiles to progeny cells [1, 3, 4].

HMTs can be grouped into two divergent families; histone lysine methyltransferases (HKMTs) catalyze the methylation of lysine residues, and protein arginine methyltransferases (PRMTs) catalyze the methylation of arginine residues. HKMTs are conserved in a wide range of eukaryotes, playing roles in cellular signaling pathways related to the cell cycle, transcription, and pathogenesis [1, 5, 6]. In* Fusarium graminearum*, H3K4me is required for the active transcription of genes involved in deoxynivalenol and aurofusarin biosynthesis [7]. The histone H3K4 methyltransferase FgSET1 plays important roles in the response to agents that damage the cell wall by negatively regulating the phosphorylation of FgMgv1 [7, 8]. Another H3K27me3 methyltransferase, KMT6, regulates the development and expression of secondary metabolite gene clusters in* F. graminearum *[9].

The SET domain is an evolutionarily conserved domain found in HMTs. In* Neurospora crassa*, the SET domain-containing protein set-2 encodes a histone H3K36 methyltransferase and is essential for normal growth and development [10–12]. The* Drosophila* trithorax group protein absent, small, or homeotic discs 1 (Ash1) contains a SET domain and is involved in maintaining active transcription of many genes by conferring a relaxed chromatin structure [13–20]. In mammals, Ash1-like (ASH1L) is the Ash1 homolog and occupies most actively transcribed genes and methylates histone H3 in a nonredundant fashion at a subset of genes, including Hox genes [13, 17, 20, 21]. In human HeLa cells, Ash1 methylates histone H3 at lysine 36 and regulates the development of myelomonocytic lineages from hematopoietic stem cells [22]. However, roles for the fungal Ash1 ortholog, a well-studied H3K36me2 methyltransferase, have not yet been extensively explored in* M. oryzae*.

*M. oryzae *is a causal agent of rice blast disease, which is the most devastating and persistent disease in cultivated rice [23, 24].* M. oryzae* can produce a three-celled asexual spore called a conidium as its primary source of inoculum. When a conidium lands on the surface of a leaf from a rice plant, it germinates to form an appressorium as the infection structure. The appressorium can enter into the host plant and grow inside the plant tissues, leading to yield losses or even death of the infected plant [25–28]. Recently, several studies have focused on the molecular mechanism of HKMTs in plant pathogenesis and plant infection [7, 8, 29]. The* M. oryzae* histone H3K4me3 methyltransferase gene* SET1 *was targeted for gene disruption by homologous recombination [28]. Chromatin immunoprecipitation-sequence analysis indicated that MoSET1 can regulate global gene expression during infection-related morphogenesis [28]. Widely conserved MoKMT2H in ascomycete fungi is a functional homolog of Ash1, which is implicated in H3K4 and H3K36 methylation. In a previous study, researchers used a wheat-infecting strain to confirm that the* MoKMT2H* gene is involved in infection-related morthogenesis and pathogenicity to wheat and barley cultivars other than rice [28]. Here, we targeted the Ash1-like* MoKMT2H* gene for gene replacement to further determine the function of MoKMT2H in a rice-infecting* M. oryzae* strain. The Δ*MoKMT2H* null mutant has no obvious defect in vegetative hyphal growth, conidium morphology, conidiation, or disease lesion formation on rice and wheat leaves. However, the Δ*MoKMT2H* null mutant was delayed for conidium germination and consequently had decreased virulence. This study offers evidence for the involvement of the histone methyltransferase function of Ashl-like proteins during plant pathogenesis and may provide important implications for discovering new HKMTs in the rice blast fungus and perhaps other pathogenic plant fungi.

## 2. Materials and Methods

### 2.1. Antibodies

Antibodies against histone H3 (Abcam, ab1791), H3K36me3 (Abcam, ab9095), H3K36me2 (Abcam, ab9049), H3K4me3 (Abcam, ab8580), H3K27me3 (Upstate, 07-449), and H3K9me3 (Millipore, 07-442) were purchased commercially.

### 2.2. Fungal Strains and Growth Conditions

The wild-type strain P131 of* Magnaporthe oryzae* was used in this study. P131 was the field isolate [29], which was used to generate mutant strains. The wild-type strain P131 and all the transformant strains generated in this study were listed in Table 1. They were grown on oatmeal tomato agar (OTA) medium and cultured at 25°C under light conditions [29–31]. For extraction of genomic DNA and protein and isolation of protoplasts, fresh mycelia were braked and cultured in liquid complete medium (CM: 0.6% yeast extract, 0,3% enzymatic casein hydrolysate, 0.3% acidic casein hydrolysate, and 1% glucose) and shaken at 150 rpm at 25°C for 36 h. To determine fungal growth, mycelial plugs of  ~5 mm in diameter were inoculated on OTA plates. Colony diameter on each plate was measured for ~3 days. Each experiment was repeated three times.

### 2.3. Molecular Manipulations with Nucleic Acids

For genomic DNA extraction, genomic DNA was isolated using the cetyltrimethylammonium bromide protocol as described [31, 32]. ~1 to 2 g mycelia was harvested and ground into a fine powder and then extracted with 15 mL of extraction buffer (50 mM Tris-HCl, 100 mM EDTA, and 150 mM NaCl). Add 750 *μ*L 20% SDS, mix well, and incubate at 37°C for 1 h. Add 2.25 mL 5 M NaCl and 2 mL CTAB/NaCl buffer (10% CTAB, 0.7 M NaCl), mix well, and then incubate at 65°C for 30 min. Add chloroform : isoamyl alcohol (24 : 1) and mix well. Cell debris was removed by centrifugation at 10,000 rpm for 15 min and genomic DNA was pelleted by isopropyl alcohol. The pellet was washed with 70% alcohol and dissolved in sterilized-distilled water and stored in −20°C. For Southern blot analysis, the extracted genomic DNA was completely digested by the restriction enzyme* Eco*RI digestion, and agarose gel separation and DNA gel blotting were performed following the standard protocols [29, 30, 32]. Hybridization was performed in solution containing 6x SSC, 5x Denhardt's solution, 0.5% SDS, and 100 *μ*L mL^−1^ denatured salmon sperm DNA, at 65°C. Blots were exposed to phosphorimager analyzer (BAS-2040, Fuji Photo Film Co., Ltd., Tokyo, Japan) and visualized by phosphorimager analyzer software.

### 2.4. Generation of the* MoKMT2H* Gene Replacement Strains with Split-PCR Strategy

For generating the* MoKMT2H* gene replacement stains, target gene replacement was carried out using the split-PCR strategy [33]. The primers used to amplify the flanking sequences from the genomic DNA of P131 in this study are listed in Table 2. For the first round of PCR, the upstream and downstream flanking sequences were amplified with the primer pairs LBCK/LB-R and RB-F/RBCK, respectively. For the second round of PCR, the fused 2937-HYG DNA flanking fragments of the left arm and right arm were amplified from the DNA products of the first-round PCR with the primers LB-F/HYG-R1 and RB-R/HYG-F1, respectively. Protoplasts were isolated and the two flanking sequences were transformed into P131 protoplasts using the classic method [32, 33]. For selecting hygromycin-resistant or neomycin-resistant transformants, CM plates were supplemented with 250 *μ*g mL^−1^ hygromycin B (Roche, USA) or 400 *μ*g mL^−1^ neomycin (Ameresco, USA). The selected neomycin-resistant transformants were subjected to PCR with primers UPF/HYG-R and UPR/HYG-F, respectively, and Southern blot analysis.

### 2.5. Protein Extraction and Western Blot Analysis

For all test strains, fresh mycelia were disturbed finely and transferred to liquid CM and shaken at 150 rpm at 25°C for 36 h. The resulting mycelia (~1.5 g) were harvested and frozen in liquid nitrogen. The frozen mycelia were ground into a fine powder and transferred to 10 mL of protein extraction buffer [50 mM Tris-HCl, pH 7.5, 100 mM NaCl, 5 mM ethylene diamine tetraacetic acid (EDTA), 1% triton X-100, and 2 mM phenylmethylsulfonyl fluoride (PMSF)] and 10 *μ*L of protease inhibitor cocktail (Roche, shanghai, China) as described [8]. After homogenization with a vortex shaker, the resulting lysate was centrifuged at 12,000 ×g for 30 min at 4°C. The supernatant was then separated by 13% denaturing polyacrylamide gel (SDS-PAGE), transferred to Immobilon-P transfer membrane (Millipore, Billerica, MA, USA) with a Bio-Rad electroblotting apparatus, and evaluated using the antibodies anti-histone H3, anti-H3K36me3, anti-H3K36me2, anti-H3K4me3, anti-H3K27me3, and anti-H3K9me3, respectively [8]. Incubation with a secondary antibody (Santa Cruz, CA, USA) and chemiluminescent detection were performed as described previously [8, 29].

### 2.6. Plant Virulence Test and Observation of Infection

For the infection assay,* M. oryzae* conidia were collected from 7-day-old OTA plates and resuspended to ~2 × 10^4^ conidia/mL in 0.025% Tween-20 solution. Four-week-old seedlings of rice (*Oryzae sativa*) cv. Lijiangxintuanheigu and 8-day-old seedlings of barley (*Hordeum vulgare*) cv. E9 were used for spray infection assays [29, 30]. Plant incubation was performed as described previously [29, 30]. Conidia were sprayed onto the barley or rice leaves, which were then incubated in a moist and dark chamber at 28°C. At 9 h and 36 h after inoculation, the leaf samples were observed by microscopy. Lesion formation was examined 7 days after inoculation. For mycelium plug inoculation, the mycelium plugs of the Δ*MoKMT2H* null mutants and the wild-type strain were inoculated onto the abraded rice plants leaves and barley leaves. For conidia droplet inoculation, conidia suspended to ~2 × 10^5^ conidia/mL were inoculated onto the abraded rice plants leaves. Lesions were examined and photographed 5–7 days after inoculation. The mean number of lesions formed on 5 cm leaf tips was determined as described previously [29, 30].

## 3. Results

### 3.1. Target Gene Replacement of* MoKMT2H *in* M. oryzae*

**Figure 1:**
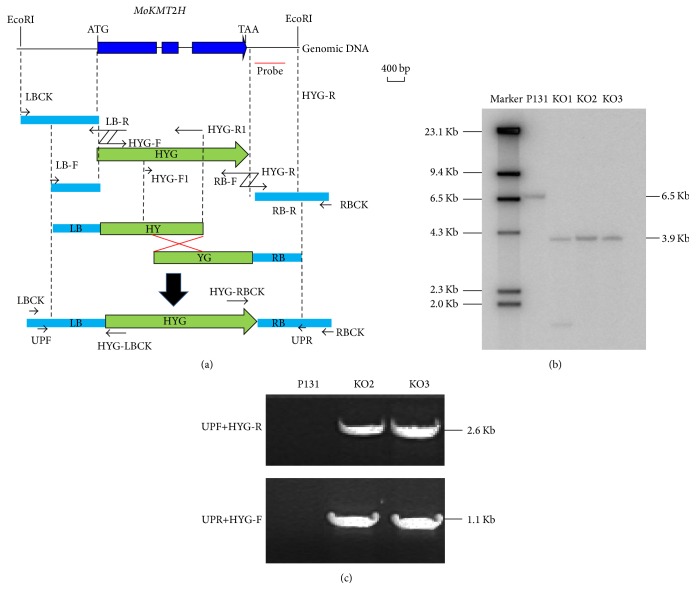
The strategy for* MoKMT2H *gene replacement with PCR. (a) Schematic representation of the genomic DNA of* MoKMT2H*. Blue boxes indicate exons. The ATG start codon and TAA stop codon are indicated. The* Eco*RI restriction enzyme sites are indicated. The red line labeled “probe” shows the region used for Southern blot analysis. The upstream and downstream flanking sequences were amplified with primer pairs LBCK/LB-R and RBCK/RB-F, respectively. The fused DNA fragments with HYG were amplified with LB-F/HYG-R1 and HYG-F1/RB-R. The DNA fragments used for transformants were amplified with LB-F/RB-F. (b) Southern blot analysis of* Eco*RI-digested genomic DNA from wild-type P131 and the neomycin-resistant transformants KO1, KO2, and KO3. Blots were hybridized with probe as indicated in (a). The DNA fragment used for a probe was amplified with primer pair Pr2937F/Pr2937R. (c) The transformants KO2 and KO3 were confirmed by amplification with primer pairs UPF/HYG-R and UPR/HYG-F.

MoKMT2H is widely conserved in ascomycete fungi. MoKMT2H is similar to mammalian Ash1l protein, which is required for H3K36me2 methylation. In previous studies, the Δ*MoKMT2H* mutants showed a significant reduction in vegetative growth, germination, appressorium formation, and pathogenicity to host plants [25]. However, the researchers used a wheat-infecting* M. oryzae* strain in their experiments and their tested* M. oryzae* strain cannot infect rice. To further determine the function of MoKMT2H in a rice-infecting* M. oryzae* strain, we performed target gene replacement of* MoKMT2H* with a hygromycin phosphotransferase cassette using the split-marker recombination method (Figure 1(a)). Three neomycin-resistant transformants, KO1, KO2, and KO3, were selected for verification by PCR or Southern blot analysis. To further characterize the* MoKMT2H* gene deletion in the selected strains KO1, KO2, and KO3, we performed Southern blot analysis using a DNA fragment as a probe. The whole genomes of KO1, KO2, and KO3 were digested with* Eco*RI. When probed with a 0.3-kb DNA fragment, amplified with primer pairs Pr2937F/Pr2937R (Table 2), the Δ*MoKMT2H* null mutants produced a 3.9-kp band, whereas the wild-type strain had a 6.5-kp band (Figure 1(b)). For PCR verification, we used two primers, UPF/HYG-R and HYG-F/UPR, in our study. When amplified with primer pair UPF/HYG-R, the Δ*MoKMT2H* but not wild-type P131 strain had a 2.6-kb DNA band, whereas the Δ*MoKMT2H* strain had a 1.1-kb DNA band with primers HYG-F/UPR (Figure 1(c)). These results confirm that the* MoKMT2H* gene was completely replaced.

### 3.2. *MoKMT2H* Is Required for Conidium Formation and Pathogenicity on Wound Leaves of Rice

**Figure 2:**
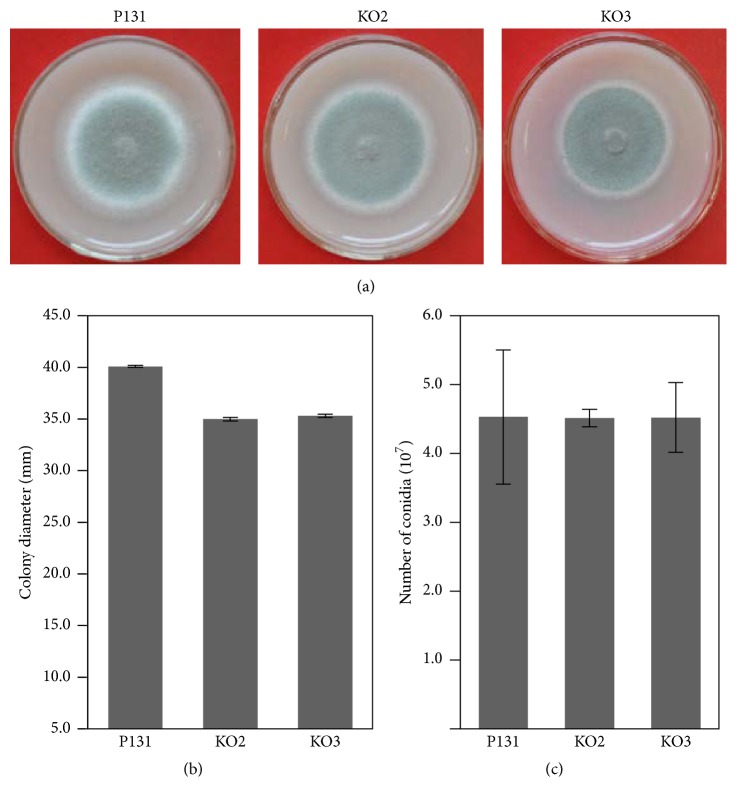
Comparisons of colony morphology, hyphal growth rate, and conidiation capacity of wild type (P131) and the gene deletion mutants of MoKMT2H. (a) Colony morphology of each strain incubated on OTA plates for 5 days at 25°C. (b) Colony diameter of each strain. (c) The average number of conidia for each strain. Values are the mean ± SD from three biological replicates.

**Figure 3:**
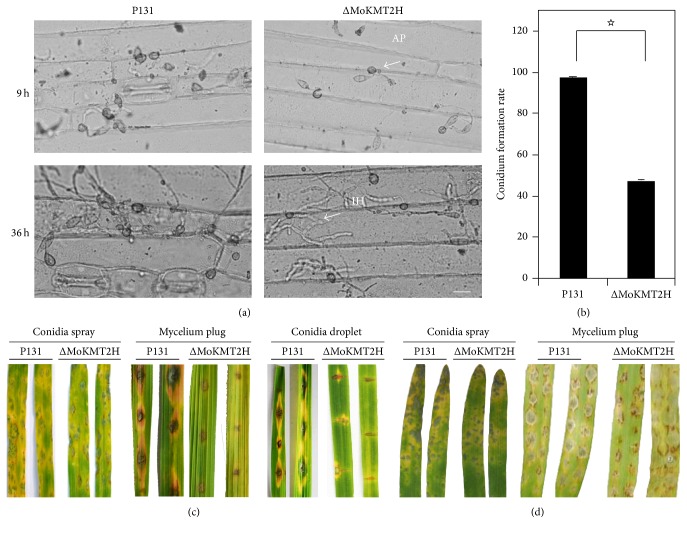
MoKMT2H is required for conidium formation and pathogenicity on rice leaves. (a) Microscopic observation of conidium formation in the wild-type strain and ΔMoKMT2H null mutants on barley leaves at 9 and 36 h after inoculation. Bar 20 *μ*m. AP: appressorium, IH: infectious hyphae. (b) Conidiation of P131 and ΔMoKMT2H null mutants after growth on OTA plates for 7 days. Comparison of the conidium formation rate between P131 and ΔMoKMT2H null mutants at 48 h after conidiation. Values are the mean ± SD from three biological replicates. (c) Conidia spray (left), mycelium plug (middle), and conidia droplet inoculation on rice leaves with conidia from the wild-type P131 and the ΔMoKMT2H null mutants. Typical leaves were observed 7 days after mycelium plug inoculation. Mycelium plug and conidia droplet inoculation was conducted on abraded rice leaves. Typical leaves were observed 5 days after mycelium plug inoculation. (d) Conidia spray (left) and mycelium plug (right) inoculation on barley leaves with conidia from the wild-type P131 and the ΔMoKMT2H null mutants. ☆ indicates *P* < 0.05.

To further characterize the biological and chemical roles of* MoKMT2H *in* M. oryzae*, we compared the colony morphology, hyphal growth rate, conidiation capacity, and virulence on the leaves of rice. The Δ*MoKMT2H* null mutants and wild-type P131 were incubated on OTA plates for 3 days. The Δ*MoKMT2H* null mutants showed no obvious defects in colony morphology (Figure 2(a)), hyphal growth rate (Figure 2(b)), or the capacity for conidium formation (Figure 2(c)) in comparison with the wild-type P131 strain (Figure 2(a)), suggesting that* MoKMT2H* is not required for asexual development in* M. Oryzae. The *Δ*MoKMT2H* null mutant strain KO3 was selected for plant virulence test. To evaluate virulence of the Δ*MoKMT2H* null mutants, conidia suspensions from the Δ*MoKMT2H* null mutants and from wild-type P131 were inoculated onto the surface of barley. The Δ*MoKMT2H* conidium formation was reduced by 61% in comparison with the wild type 9 h after inoculation (Figures 3(a) and 3(b)). Strikingly, at 36 h after inoculation, wild-type P131 formed bulbous infection hyphae and could extend to neighboring host cells, whereas the Δ*MoKMT2H* null mutants had obvious defects in penetration peg formation (Figure 3(a)). To further confirm the plant infection defects in Δ*MoKMT2H* null mutants, conidia suspensions of the Δ*MoKMT2H* null mutants and wild-type P131 were sprayed onto seedlings of rice cultivar LTH. Typical robust lesions of the rice blast were observed for the wild type. However, the lesions of Δ*MoKMT2H* null mutants were not markedly reduced as compared with the wild type (Figure 3(c)). For the barley leaves, typical disease lesions were observed from both the wild type and the Δ*MoKMT2H* null mutant strains following spray inoculation of conidia (Figure 3(d)). To further check whether the Δ*MoKMT2H* null mutants could infect the host cells through wounds, the mycelium plugs of the Δ*MoKMT2H* null mutants and wild type were inoculated onto abraded rice leaves (Figure 3(c)). Although inoculation with the wild-type strain caused severe and typical lesions on wound rice leaves, the Δ*MoKMT2H* null mutants could not cause any disease lesions, suggesting that* MoKMT2H* was essential for penetration peg formation on wounded rice leaves (Figure 3(c)). To further confirm our observation, we performed conidia droplet inoculation on wounded rice plant leaves. As shown in Figure 3(c), inoculation with the wild-type caused typical disease lesions around the wound sites compared with the Δ*MoKMT2H* null mutants. Therefore, these results suggest that* MoKMT2H* regulates conidium formation and penetration peg formation in wound pathogenesis on rice leaves.

### 3.3. *MoKMT2H* Is a Conserved Set Domain-Containing Protein but Is Not Involved in Genome-Wide Histone Methylation

**Figure 4:**
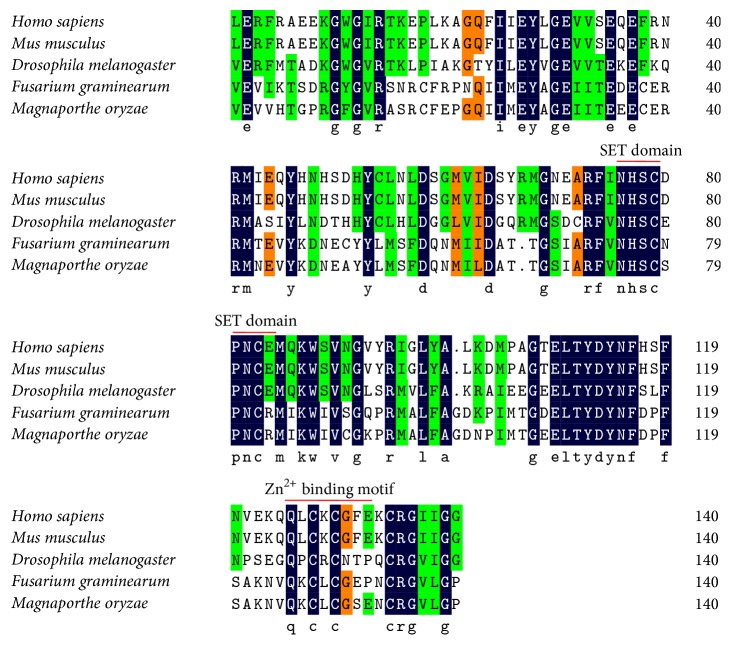
Multiple alignment of SET domain of Ashl-like proteins in classic species. The background colors indicate the conservation of amino acid residues. Orange shading color represents ≥50% similarity, green represents ≥75% similarity, and black represents 100% similarity. The conserved residues are indicated by red lines; the SET domain residues in the NHxxxPN motif and the post-SET domain, which consists of the cysteine-rich motif CxCxxxxC that binds Zn^2+^ OR that coordinates Zn^2+^ binding, are indicated.

The SET domain, which mediates lysine methylation, regulates chromatin-mediated gene transcription. Published studies show that SET proteins in lower organisms can manipulate host transcription machinery in host-pathogen interactions [10, 11]. MoKMT2H is an Ashl-like protein [12]. Ash1-like orthologs of MoKMT2H are also present in* Drosophila*,* Homo sapiens*,* Mus musculus*,* F. graminearum*, and* M. oryzae* [10–16]. To further identify the homologs of Ash1-like proteins, we assessed the conservation of the SET domains through amino acid sequence alignment (Figure 4). The sequence alignment in Figure 4 highlights the conservation of the SET domain residues in the NHxxxPN motif and the post-SET domain containing the Zn^2+^-binding motif (CxCxxxxC) among these selected Ash1-like proteins. Collectively, these data indicate that MoKMT2H is highly conserved across lower eukaryotes to higher animals, which suggests that these SET domain-containing proteins may have evolved important roles in manipulating chromatin methylation-mediated gene transcription.

**Figure 5:**
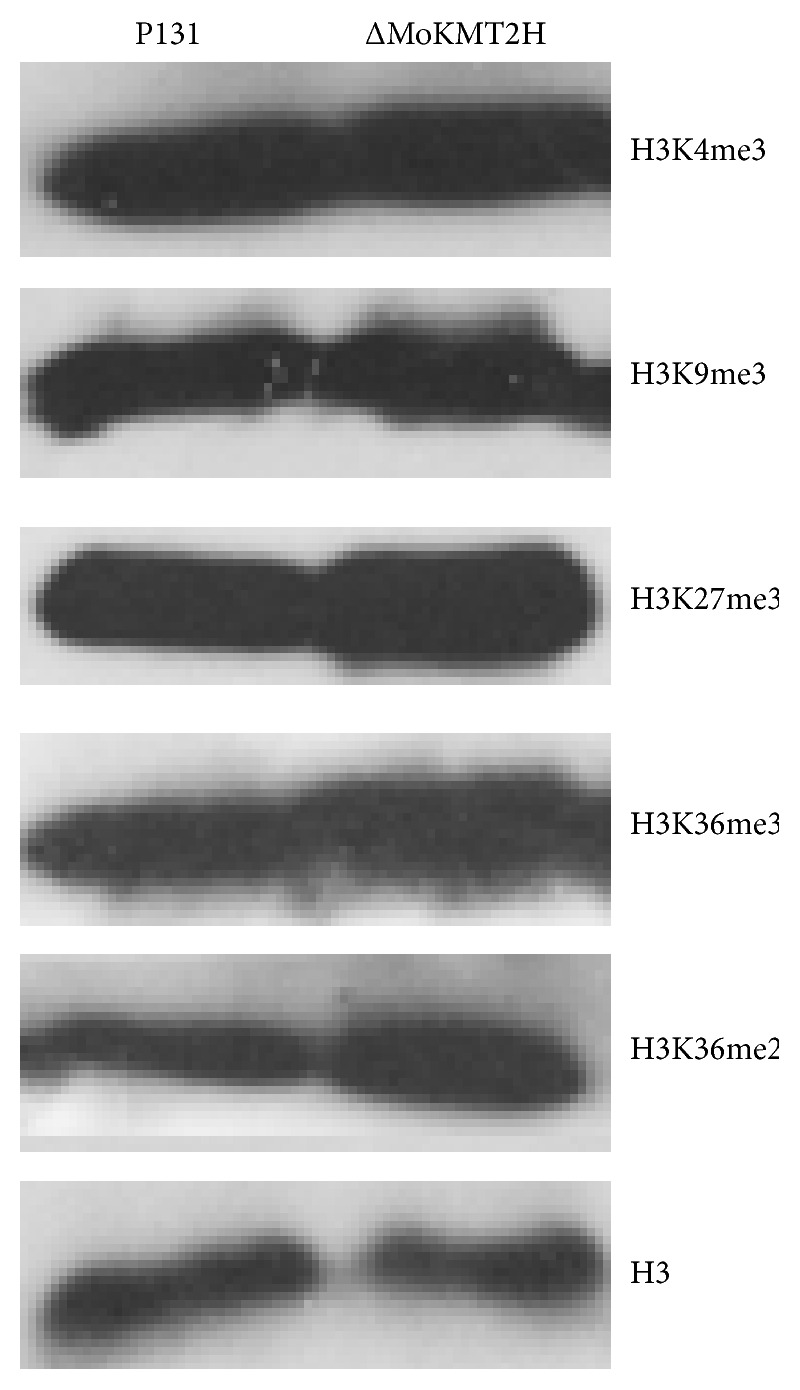
Western blot analysis of multiple histone modifications in the wild type and ΔMoKMT2H null mutants. Total proteins were extracted and separated by 13% SDS-PAGE, followed by immunoblotting with the indicated antibodies. Anti-H3 was used as a loading control.

Considering that MoKMT2H is a candidate histone methyltransferase, we decided to apply western blot analysis to determine whether the methylation pattern of genome-wide histone lysines in the Δ*MoKMT2H* null mutants was changed. Some groups reported that MoKMT2H is not specific for H3K4, H3K27me3, or H3K20me3 methylation in their KMT null mutants [28]. However, some groups proved that the SET domain of MoKMT2H is much more closely related to H3K36me2-specific methyltransferases than H3K4-specific methyltransferases such as Set1 or trithorax group proteins [20, 22]. Total proteins were extracted from the mycelia of Δ*MoKMT2H* null mutant and wild-type strains. There was no significant reduction in the signal of H3K4me3, H3K9me3, H3K27me3, and H3K36me3 methylation in the Δ*MoKMT2H* null mutant (Figure 5). Moreover, the Δ*MoKMT2H* null strain also displayed no activity on H3K36me2 of the genome-wide chromatin. It is possible that MoKMT2H may act only on some specific target genes during the process of pathogenesis.

## 4. Discussion

The SET domain, which mediates lysine methylation, is one of the major epigenetic marks and regulates chromatin-mediated gene transcription [10, 12, 17]. The multiple alignment depicts the evolutionary history of the conserved SET domain of MoKMT2H, an Ash1-like protein. The presence of the cysteine-rich (CxCxxxxC) post-SET domain facilitates the binding of Zn^2+^ ions and marks an evolutionary divergence from the homologs in other organisms. In addition, the *S*-adenosylmethionine cofactor-binding site and the post-SET motif-containing region are also highly conserved. In our study, we did not, however, detect a change in genome-wide histone methylation on H3K36me2/3, H3K4me3, H3K9me3, or H3K27me3 in the Δ*MoKMT2H* null strains (Figure 5 and data now shown), which suggests that MoKMT2H may have methylation activity only on specific target genes of histone H3 or other key regulated proteins during the process of plant pathogenesis. Further experiments will focus on the HMT activity on recombinant nucleosomes or core histones with recombinant MoKMT2H SET domain deletions in vitro.

*MoKMT2H* is required for conidiation, conidium germination, and appressorium formation [26, 28, 29], and Δ*MoKMT2H* null strains are defective for pathogenicity on wheat cultivar Norin 4 [28]. However, those previously tested wild-type and null mutant strains can only infect wheat. To further determine the biological and chemical activity of MoKMT2H, we generated Δ*MoKMT2H* null strains from the P131 wild-type strain, which forms classical lesions on rice leaves. The most interesting conclusion in this study we obtained when compared with previous studies is that deletion of* MoKMT2H* reduced the rate of conidium formation and plant virulence on wounded rice leaves. More interestingly, spray inoculation of the Δ*MoKMT2H* null strains did not cause any reduction in pathogenicity on rice leaves (Figures 3(c) and 3(d)). These results suggest that MoKMT2H may recognize specific effectors induced by wounds during the plant-pathogen interaction. Our study has thus shown that the Ash1-like protein MoKMT2H is required for conidium germination and pathogenesis in* M. oryzae*. MoKMT2H is widely conserved in ascomycete fungi; however, its biological roles have not been uncovered. Further research will focus on determining whether MoKMT2H has the chemical activity of a histone methyltransferase and isolating the proteins that it interacts with and its downstream target genes during pathogenesis.

## Figures and Tables

**Table 1:** Fungal strains used in this study.

Strain	Description	Reference
P131	The wild-type strain	Peng and Shishiyama (1988) [29]
KO1	The deletion mutant of *MoKMT2H*	This study
KO2	The deletion mutant of *MoKMT2H*	This study
KO3	The deletion mutant of *MoKMT2H*	This study

**Table 2:** PCR primers used in this study.

Primer	Sequence (5′-3′)
LBCK	GCCTGTCTGATTGGA

RBCK	GGGAGGGGGTGATGACGGTC

LB-F	CTGCTGCTTAGGTCGGTAGTCT
LB-R	TTGACCTCCACTAGCTCCAGCCAAGCCATTGGCTGGTTGGTTTGTTGGT

RB-F	GAATAGAGTAGATGCCGACCGGGCGTCACATGCGAACAAGAACCA
RB-R	GGCAAGGCAAGATTGGCTAAGA

HYG-F	CTTGGCTGGAGCTAGTGGAGGT
HYG-R	CCCGGTCGGCATCTACTCTATTC

HYG-F1	CGTTGCAAGACCTGCCTGAA
HYG-R1	GGATGCCTCCGCTCGAAGTA
HYG-LBCK	GACAGACGTCGCGGTGAGTT
HYG-RBCK	TCTGGACCGATGGCTGTGTAG
UPF	GAGAACTCAA GCGTCACTCC
UPR	GAACCAAAAGCATGTTTCT
Pr2937F	CCTTGCCTGTCTGATTGG
Pr2937R	GGAGTGACGCTTGAGTTC

## References

[1] Kouzarides T. (2007). Chromatin modifications and their function. *Cell*.

[2] Margueron R., Trojer P., Reinberg D. (2005). The key to development: interpreting the histone code?. *Current Opinion in Genetics and Development*.

[3] Grunstein M. (1997). Histone acetylation in chromatin structure and transcription. *Nature*.

[4] Martin C., Zhang Y. (2005). The diverse functions of histone lysine methylation. *Nature Reviews Molecular Cell Biology*.

[5] Black J. C., Van Rechem C., Whetstine J. R. (2012). Histone lysine methylation dynamics: establishment, regulation, and biological impact. *Molecular Cell*.

[6] Greer E. L., Shi Y. (2012). Histone methylation: a dynamic mark in health, disease and inheritance. *Nature Reviews Genetics*.

[7] Jeon J., Choi J., Lee G.-W. (2015). Genome-wide profiling of DNA methylation provides insights into epigenetic regulation of fungal development in a plant pathogenic fungus, *Magnaporthe oryzae*. *Scientific Reports*.

[8] Liu Y., Liu N., Yin Y., Chen Y., Jiang J., Ma Z. (2015). Histone H3K4 methylation regulates hyphal growth, secondary metabolism and multiple stress responses in *Fusarium graminearum*. *Environmental Microbiology*.

[9] Connolly L. R., Smith K. M., Freitag M. (2013). The *Fusarium graminearum* histone H3 K27 methyltransferase KMT6 Regulates development and expression of secondary metabolite gene clusters. *PLoS Genetics*.

[10] Qian C., Zhou M.-M. (2006). SET domain protein lysine methyltransferases: structure, specificity and catalysis. *Cellular and Molecular Life Sciences*.

[11] Bannister A. J., Schneider R., Kouzarides T. (2002). Histone methylation: dynamic or static?. *Cell*.

[12] Nwasike C., Ewert S., Jovanovic S., Haider S., Mujtaba S. (2016). SET domain-mediated lysine methylation in lower organisms regulates growth and transcription in hosts. *Annals of the New York Academy of Sciences*.

[13] Byrd K. N., Shearn A. (2003). ASH1, a *Drosophila* trithorax group protein, is required for methylation of lysine 4 residues on histone H3. *Proceedings of the National Academy of Sciences of the United States of America*.

[14] Gregory G. D., Vakoc C. R., Rozovskaia T. (2007). Mammalian ASH1L is a histone methyltransferase that occupies the transcribed region of active genes. *Molecular and Cellular Biology*.

[15] Shearn A. (1989). The ash-1, ash-2 and trithorax genes of *Drosophila melanogaster* are functionally related. *Genetics*.

[16] Klymenkoand T., Müller J. (2004). The histone methyltransferases Trithorax and Ash1 prevent transcriptional silencing by Polycomb group proteins. *EMBO Reports*.

[17] Papp B., Müller J. (2006). Histone trimethylation and the maintenance of transcriptional ON and OFF states by trxG and PcG proteins. *Genes and Development*.

[18] Schwartz Y. B., Kahn T. G., Stenberg P., Ohno K., Bourgon R., Pirrotta V. (2010). Alternative epigenetic chromatin states of polycomb target genes. *PLoS Genetics*.

[19] Beisel C., Imhof A., Greene J., Kremmer E., Sauer F. (2002). Histone methylation by the *Drosophila* epigenetic transcriptional regulator Ash1. *Nature*.

[20] Tanaka Y., Katagiri Z.-I., Kawahashi K., Kioussis D., Kitajima S. (2007). Trithorax-group protein ASH1 methylates histone H3 lysine 36. *Gene*.

[21] Schwartz Y. B., Kahn T. G., Stenberg P., Ohno K., Bourgon R., Pirrotta V. (2010). Alternative epigenetic chromatin states of polycomb target genes. *PLoS Genetics*.

[22] Yuan W., Xu M., Huang C., Liu N., Chen S., Zhu B. (2011). H3K36 methylation antagonizes PRC2-mediated H3K27 methylation. *The Journal of Biological Chemistry*.

[23] Wilson R. A., Talbot N. J. (2009). Under pressure: investigating the biology of plant infection by *Magnaporthe oryzae*. *Nature Reviews Microbiology*.

[24] Saunders D. G. O., Aves S. J., Talbot N. J. (2010). Cell cycle-mediated regulation of plant infection by the rice blast fungus. *Plant Cell*.

[25] Skamnioti P., Gurr S. J. (2009). Against the grain: safeguarding rice from rice blast disease. *Trends in Biotechnology*.

[26] Talbot N. J. (2003). On the trail of a cereal killer: exploring the biology of *Magnaporthe grisea*. *Annual Review of Microbiology*.

[27] Gilbert M. J., Thornton C. R., Wakley G. E., Talbot N. J. (2006). A P-type ATPase required for rice blast disease and induction of host resistance. *Nature*.

[28] Minh Pham K. T., Inoue Y., Van Vu B. (2015). MoSET1 (Histone H3K4 methyltransferase in *Magnaporthe oryzae*) regulates global gene expression during Infection-related morphogenesis. *PLOS Genetics*.

[29] Peng Y. L., Shishiyama J. (1988). Temporal sequence of cytological events in rice leaves infected with *Pyricularia oryzae*. *Canadian Journal of Botany*.

[30] Yang J., Kong L., Chen X. (2012). A carnitine-Acylcarnitine carrier protein, MoCrc1, is essential for pathogenicity in *Magnaporthe oryzae*. *Current Genetics*.

[31] Xu J.-R., Hamer J. E. (1996). MAP kinase and cAMP signaling regulate infection structure formation and pathogenic growth in the rice blast fungus *Magnaporthe grisea*. *Genes and Development*.

[32] Yang J., Zhao X., Sun J. (2010). A novel protein com1 is required for normal conidium morphology and full virulence in *Magnaporthe oryzae*. *Molecular Plant-Microbe Interactions*.

[33] Goswami R. S., Bolton M. D., Thomma B. P. H. J. (2012). Targeted gene replacement in fungi using a split-marker approach. *Plant Fungal Pathogens: Methods and Protocols*.

