# Corrigendum to “An Ash1-Like Protein MoKMT2H Null Mutant Is Delayed for Conidium Germination and Pathogenesis in Magnaporthe oryzae”

**DOI:** 10.1155/2019/7639476

**Published:** 2019-07-04

**Authors:** Zhaojun Cao, Yue Yin, Xuan Sun, Jun Han, Qing peng Sun, Min Lu, Jinbao Pan, Weixiang Wang

**Affiliations:** Beijing Key Laboratory of New Technique in Agricultural Application College of Plant Science and Technology, Beijing University of Agriculture, Beijing 100026, China

In the article titled “An Ash1-Like Protein MoKMT2H Null Mutant Is Delayed for Conidium Germination and Pathogenesis in Magnaporthe oryzae” [1], there was a spelling error in the first sentence in the Abstract. “Ash1 is a known H3K36-specific histone demethylase that is required for normal Hox gene expression and fertility in Drosophila and mammals.” should be updated to “Ash1 is a known H3K36-specific histone methylase that is required for normal Hox gene expression and fertility in Drosophila and mammals.”
